# General Paralysis of the Insane

**Published:** 1854-01-01

**Authors:** 


### Art. IV.—GENERAL PARALYSIS OF THE INSANE.

It is a singular fact that, notwithstanding the deeply interesting nature of the subject, only one monograph on the General Paralysis of the Insane (the treatise by Dr. Winn) has appeared in this country; whilst on the continent, not less than five monographs have been published during the last thirty years. Dr. Winn's treatise, published in 1848, and previously printed as an original article in the "Psychological Journal," contained a full account of all that was known respecting the causes, symptoms, progress, and pathology of this peculiar disease. Since then (1848), three fresh works on general paralysis have issued from the French press. The most recent of these, from the pen of M. Jules Falret* (son of the well-known physician to the Salpêtrière), contains a good digest of all that has been written of late on the subject, and as a methodical and comprehensive treatise it will be found of service; the practical physician will, however, regret to notice that the author's speculative remarks are more numerous than his clinical observations.

Much confusion and a variety of conflicting opinions prevail on the continent respecting the nature of general paralysis, and it is not easy to determine, from the statements of foreign writers, what they precisely mean by their various descriptions of the disease. M. Falret attempts to clear up the difficulty. He says:

"Relative to general paralysis, there are four leading opinions: one, the most ancient, advocated by Delaye, Calmeil, Georget, and also by Esquirol and the greater number of his élèves, implies that the disease is a simple complication or termination of any kind of insanity; another opinion, supported by Bayle, Parchappe, and Ducheck, of Prague, represents the affection as a distinct special form of mental disease, characterized at the same time by physical as well as mental symptoms, and by anatomical lesions; a third notion, conceived by Requien, Baillarger, Lunier, and Rodrigues, confounds, by means of one symptom, that of paralysis, those cases which are, and those which are not, attended with delusion, under the name of *progressive general paralysis;* lastly, the fourth view, supported by Sandras, Brierre de Boismont, and Duchenne du Boulogne, recognises two principal kinds of general paralysis; one, in which the paralysis is associated with insanity; the other, in which the paralytic symptoms are unconnected with any signs of mental aberration."

M. Falret follows up these statements by a query—

"Are there two kinds of general paralysis, the one *with*, the other *without*, insanity, or is there only one form of the disease?"

* Recherches sur la Folie Paralytique et les Diverses Paralysies Générale. Par Jules Falret, Docteur en Médecine, ancien Interne des Hôpitaux et Hospices Civils de Paris. 1853.

This point, he thinks, might be determined by a comparison of the cases in asylums for the insane with those in ordinary hospitals. It would however appear, from the cases which he has appended to his treatise, and to which we shall have occasion to allude, that the experience to be gained from general hospitals, with respect to general paralysis, is of the scantiest and most inconclusive kind. M. Falret is forced to admit that, in our present state of knowledge, it is impossible to decide whether there is a form of general paralysis which may exist independent of insanity, and he contents himself with an inquiry into the nature of the general paralysis of the insane, and to which he gives the name of paralytic insanity, but which we think less definite than the term in common use. We shall, however, in this review, adopt the author's designation, as it may help to define his particular notion of the disease.

M. Falret divides his treatise into two parts. In the first, he endeavours to show that paralytic insanity is a special form of disease, characterized not only by anatomical lesion and by the special phenomenon of the paralysis, but also by its psychical symptoms and by its peculiar mode of progression. In the second part he takes a rapid review of the various maladies which might be confounded with paralytic insanity, in order to discriminate between them and the particular malady more especially under consideration.

M. Falret, *en passant*, moots the following question :—"Is there," he says, "another and totally distinct form of paralysis, which would merit the term of *progressive?*" This point, he thinks, cannot be determined in the present state of our knowledge.

The following remarks by the author are extremely judicious :—

"It is not necessary to establish the fact that paralysis precedes, in nearly every instance, the accession of mental derangement, in order to prove that paralytic madness is a special affection and not a termination of every kind of insanity. In fact, the proposition advanced by MM. Baillarger and Lunier does not appear to be generally true. They have advocated the anteriority of the symptoms of paralysis ; our observations, on the other hand, would lead us to suppose, as a general rule, that the mental derangement and the paralysis have a simultaneous origin, although in many instances the affection of the mind precedes, for a longer or shorter period, the manifestation of the paralysis."

M. Falret concludes this part of the subject with the following propositions, which he considers incontestable :

"1st. That this specific form of paralysis is never known to attack insane patients who have been confined for many years in an asylum. If paralysis occasionally attacks patients belonging to this class, it is a totally different malady, and depends upon softening of the brain, apoplexy, tumours of the brain, or upon affections perfectly distinct from paralytic insanity.

"2ndly. That the insane who die paralytic in asylums, invariably exhibit traces of paralysis on their admission, or very soon afterwards, and that none of these patients survive a period of more than three or four years."

We can vouch for this high rate of mortality, which is rather below than under the truth. Out of 90 cases that were alive twelve months since in one of our large asylums, scarcely a ninth part are now alive.

M. Falret does not profess, in his treatise, to give a detailed description of the course of paralytic insanity; his object is to prove, that in spite of individual differences, the disease presents a generic character. He justly observes that a great obstacle attends an investigation into the nature of the disease at its outset, owing to the difficulty of obtaining satisfactory information in the early periods of its manifestation. The approach of the disease is so insidious that it often makes considerable progress without the relatives of the patients having been in the slightest degree aware of its existence. It is, generally speaking, only at a late period, when the symptoms are sufficiently evident, that the medical attendant has an opportunity of witnessing the progress of the complaint.

M. Falret recognises four varieties of paralytic insanity:—the *congestive*, the more especially *paralytic*, the *melancholic*, and the *expansive* variety. As the author's views respecting these forms of the disease are full of interest, we shall give them at length:—

"1. *Congestive variety.*—Congestion has often been noticed as a precursory symptom of general paralysis, but its importance has been exaggerated. M. Bayle, for instance, asserts that the disease is always marked by giddiness and *d'afflux de sang à la tête*, in addition to more or less impairment of the reasoning faculties. It must be understood that this congestive state has no reference to those sudden attacks of congestion of the brain and of epilepsy which occasionally occur during the progress of the disease. It generally happens that some of the physical or psychical symptoms which characterise the affection, have existed for some time before the explosion of the congestive attack. The embarrassment of speech often becomes more intense after one of these seizures, and the mind becomes disturbed. The accompanying insanity may be marked by excitement or depression.

"2. *Paralytic variety.*—So little is known respecting this form of the disease, that its demonstration is by no means an easy task. It is this very paucity of facts which has given rise of late to so much controversy with regard to the nature of the malady. Occasionally, in ordinary practice, we meet with patients who, without any known cause, exhibit an almost imperceptible tremor of the upper extremities, a difficulty in performing delicate manipulations with the fingers, such as writing, for instance, an irregularity of gait, a difficulty of supporting themselves on their legs, and a trifling amount of hesitation in speaking. They seem conscious of these defects, and attribute them to some accidental or permanent cause, such as fatigue, cold, or the loss of a tooth. Tremor of the tongue and upper lip are also frequently observable. Vertigo is also a common symptom. Sometimes, as M. Baillarger observes, there is irregular dilatation of the pupils, and impairment of the sexual functions. In general, these paralytic patients appear to be in full possession of their intellectual faculties; but, if they are closely examined, and above all, if the persons who live with them are questioned minutely, we discover signs of impaired reason, even in the first stages of the disease. Very soon, however, the mental disorder becomes fully developed. The habits of the patients are completely changed, and they perform a variety of singular acts, quite inexplicable to those who are acquainted with their previous mode of life.

"The duration of this *état prodromique* is necessarily variable, and may be prolonged for a considerable period. The paralytic phenomena increase progressively. The intelligence becomes weakened, and at length decided symptoms of insanity are manifested. The patient may be either in a state of excitement or depression, but in nearly every instance the mental disorder is marked by ideas of grandeur or by self-satisfaction.

"3. *Melancholic variety.*—It is generally supposed, when paralytic insanity commences with mental disorder, that it invariably gives rise to a change of habits and character, and is accompanied by great mental and physical excitement; yet if we inquire minutely into the history of the case, we shall occasionally learn that the patient, before the accession of maniacal excitement, had been in a state of moral depression, and to which some authors, and especially Dr. Ducheck, have given the name of *le stade mélancolique.* MM. Bayle and Calmeil frequently allude to the state of melancholy which precedes the appearance of the phenomena of excitation. It is frequently marked by a short intermittence, during which the patient resumes his ordinary occupation. This interval is, however, very brief, and is quickly followed by an explosion, as it were, of agitation and incoherence.

"4. *Expansive variety.*—This form, which is the most common, has been frequently described. It may precede or follow any of the other varieties. Its commencement is marked by an excessive activity, which betrays itself in actions and in language. The patient's character is modified or exaggerated; he becomes more active in his profession, and occupies himself at the same time with new pursuits. He sleeps little, and conceives projects which are not altogether absurd, but which can only be realised up to a certain point. He gives himself up to intoxication, to venereal excesses, or to singular actions, which are often dangerous, and sometimes criminal. It is in this variety that the patient commits thefts and other felonious acts, which lead to his arrest. When the patient arrives at this stage of extreme excitement, we often see them a prey to excessive activity, which may terminate in one night, or in a few hours, in maniacal agitation, or in those delusions of grandeur that are so characteristic of the malady."

The temporary benefit obtained by medicine in this disease is often so striking as to lead medical men to suppose that they have cured cases, when speedy relapses too often show how fallacious have been their conclusions. M. Falret does not deny the possibility of a perfect cure, and he instances a case of M. Fevrier's which had not relapsed during a period of twenty-five years. He, however, believes that in many instances the supposed recoveries were merely examples of intermission. In one case of his own, the intermission lasted a year and a half. In the practice of his father, at Salpêtriere, he has frequently seen the most marked remissions follow the application of the actual cautery to the nape of the neck. M. Baillarger, in his clinical lectures, has pointed out the frequent disappearance of the delusions in the course of the disease; he, however, considers that the paralytic symptoms, and especially the embarrassment of speech, never wholly disappear, and that they evince, by their presence, the persistence of the disease. M. Falret relates, on the authority of Dr. Coindet, of Geneva, an interesting case of paralytic insanity, in which the disease, after having been remarkably intense and characteristic, was so completely suspended for a period of five months, that the medical attendant was only once able to detect a very slight embarrassment of speech, and this did not occur until the close of the intermission. The recurrence of the disease was marked by epileptiform attacks, which terminated the patient's existence at the end of a month.

It is generally admitted that the mental disorder associated with paralytic insanity, may appear under three principal forms—viz., monomania, mania, and dementia. M. Falret, however, asks this question: "Is it not possible, by penetrating beyond the external manifestations of this disease, to discover a special character in the form of insanity which accompanies general paralysis, and which distinguishes it from that of any other species of mental affection?" M. Baillarger inclines to this opinion: "The ordinary monomaniacs," he says, "differ from the paralytic monomaniacs by the obstinacy with which they retain their ideas; the paralytic, on the contrary, on account of the feebleness of their memory, have no coherence in their ideas, and they constantly contradict themselves."

M. Falret's views respecting the character of the insanity which is associated with general paralysis, is extremely faulty as regards arrangement. He has fallen into an error so common to a youthful, ardent, and imaginative Frenchman—that of unnecessarily subdividing and complicating his subject, until all idea of unity is lost. He has, for instance, divided the evolution of the insanity into three distinct phases, which he terms the period "*d'incubation ou de production des idées délirantes*," the "*période de systematisation*," and the "*période es-*

*sentiellement chronique de l'alienation.*" All this may appear very scientific, but it is decidedly unphilosophical.

We have no doubt that ambitious or exalted monomania is the form of insanity more commonly associated with paralytic insanity. Generally speaking, it is some mental eccentricity which has first attracted the attention of the patient's friends, his bodily infirmity having been quite overlooked. The patients speak of their fortune and grandeur, that they are able to make considerable purchases, and build palaces. Everything is transformed into gold or silver; and the very pebbles are precious stones, which they hoard with care. Some consider the asylum as a magnificent palace, the persons around them as only there to wait on them; and the strangers who come to visit the institution are regarded as petitioners for their powerful influence. Others consider their detention as a shameful injustice, opposed to all laws, both human and divine. They promise thousands to the physician for their release, and they seek to corrupt the keepers by signing bills for enormous sums of money. One fancies he is a king or an emperor, and exacts homage; another imagines himself to be a deity, requiring adoration. In an asylum containing patients suffering from general paralysis, we find in imagination barons, peers, generals, physicians, astronomers, poets, men who know everything, even to the secrets of Providence, and whose power is such as to give motion to the universe. In the second period of general paralysis the patients have the same ideas of grandeur, but the insanity is more general. Ambitious monomania, however, does not invariably accompany paralytic insanity. The insane aspect varies very much in different individuals, and in the same person at different times. The disease generally begins with mania or monomania, and ends in dementia.

M. Falret, in the second portion of his work, deprecates the tendency evinced by recent authors to unite under the head of general paralysis or progressive paralysis, facts which differ strikingly from each other, whether we regard the general symptoms, the progress of the malady, or the peculiar paralytic features of each individual disease. He then proceeds to notice the various diseases liable to be confounded with paralytic insanity. He begins with affections of the brain.

"*Cerebral hæmorrhage.*—When the invasion of this disease is sudden and accompanied by hemiplegia, there is little probability of its being confounded with paralytic insanity. Very often the intellect is unaffected, and the embarrassment of speech is persistent. The only cases of cerebral hæmorrhage which are likely to lead to an error of diagnosis, are those in which a less intense paralysis of one half of the body supervenes upon an old hemiplegic attack of the opposite side. This generalisation of the paralysis is generally attributed, by authors, to an effusion of serum upon the brain or into the ventricles, and which is consecutive to the primitive lesion."

"*Softening of the brain.*—The diagnosis, in some instances, between this affection and general paralysis, is much more difficult than between the latter disease and cerebral hæmorrhage. * * * The signs which especially distinguish softening of the brain from paralytic insanity, are as follows—intense and continued headache, frequent vomiting, violent pains in limbs, cramps, and occasional numbness of one side of the body. Embarrassment of speech rarely occurs, but when present it is more marked than in paralytic insanity. The intellect is generally unaffected, but the memory is frequently impaired. Anæsthesia and hyperæsthesia more commonly occur in this affection than in paralytic insanity. Moreover, the indications to be derived from the course of the disease must not be overlooked. The progress of softening of the brain is often rapid, and the invasion of the disease is frequently sudden. The paralysis and impairment of consciousness does not generally supervene until after one or more attacks. The course of the disease is much more rapid than that of general paralysis, and the patients seldom survive more than a few months."

"*Tumours of the brain.*—The principal characteristics which distinguish cerebral hæmorrhage and softening, apply with equal force to tumours of various kinds, tubercle, cancer, cysts, exostoses, syphilitic nodes, &c. * * * Tumours of the brain have ordinarily a long duration and a slower development than paralytic insanity, although the evolution of the latter disease is remarkably slow."

"*Meningitis.*—The progress of *acute* meningitis is generally so extremely rapid, that the possibility of confounding it with general paralysis is extremely slight, unless, as some authors assert, there be a form of *acute* paralytic insanity; this, however, is extremely problematical. * * * The assimilation of these two affections appears to have been owing to a mistaken identity or to anatomical lesion. It is possible that during the progress of general paralysis meningitis may ensue; should this, however, be the case, the occurrence must be merely considered in the light of an accidental complication."

"*Affections of the spinal cord.*—Very little reflection will suffice to show that this disease bears only a partial resemblance to paralytic insanity. Medullary paralysis is almost invariably paraplegic, and if general paralysis ensues it supervenes solely as a secondary affection. There is, moreover, neither disorder of the intellect nor embarrassment of speech."

"*Nervous paralysis.*—Since modern anatomical researches have discovered in the nervous centres the origin of the greater number of paralytic diseases, we are little disposed to acknowledge the existence of any affection of the nervous system which is unassociated with lesion of some part of the brain or spinal cord; nevertheless, a certain number of these maladies have been classified by the best authorities, as a distinct and peculiar species of functional disease. They may be divided into two classes—those paralytic cases which depend on epilepsy, hysteria, chorea, &c., and those which are the result of poisoning from alcohol, lead, mercury, &c."

"*Paralysis from the effects of lead.*—It is possible that persons who have been long exposed to the influence of lead, may be attacked with symptoms closely resembling paralytic insanity, but this must not be confounded with the true general lead-paralysis, which constitutes a special disease. We have met with two cases of general paralysis which appeared to have been occasioned by lead. In one, there was a complete paralysis of all the members of the body, but the case differed in several respects from one of paralytic insanity. The affection occurred in a young man who was at first attacked with paralysis of the extensor muscles of the forearms. For these symptoms, electricity and transcurrent cauterisation were employed in vain, under the direction of M. Bricheteau. A year afterwards the paralysis had extended to the thighs and the trunk of the body. He died suddenly, and a post-mortem examination revealed softening of the superior part of the brain."

"*Mercurial trembling.*—The action of mercury has been supposed to be a cause of the paralysis of the insane, but the arguments which have been brought forward in proof of this opinion are extremely questionable. Mercurial paralysis has *un cachet tout spécial*, and notwithstanding the analogy of some symptoms, the diseases cannot be considered as identical."

"*Paralysis from the effects of alcohol.*—This affection bears a closer resemblance to paralytic insanity than any of the preceding diseases. We do not allude to acute delirium tremens, but to the affection which Dr. Huss has termed alcoholismus chronicus. * * * The first phenomenon which commonly exhibits itself in those who indulged to excess in alcoholic drinks, is trembling of the hands, which is more manifest in the morning than in the evening. It is also most conspicuous after numerous excesses, or after the sudden deprivation of the stimulus to which the system has become accustomed. For a long period this trembling is, in many drunkards, the only symptom produced by the abuse of alcoholic liquors. Not unfrequently, however, they are attacked with headache, vertigo, and dazzling of the sight, which obliges the patient to lean for support on any object near at hand. The digestive organs are frequently deranged at the very outset of the affection, and a vomiting of mucus often occurs in the morning. After a while, if the abuse of alcohol be continued, we notice the first symptoms of alcoholic paralysis, and which have been so well described by Dr. Huss. Formications in the upper and lower extremities, numbness in the fingers and toes, and cramps and pains of the limbs, are amongst some of the commonest signs of the disease. All these phenomena of muscular and nervous weakness are generally confined to the extremities, and seldom extend above the neck. At the same time the brain becomes the seat of abnormal symptoms, indicated by giddiness, indistinct vision, and muscæ volitantes. * * * Many symptoms of chronic alcoholism resemble general paralysis at their outset. The former disease, however, differs from the latter by the fact of the paralysis generally commencing with a numbness of the toes and fingers before these organs become decidedly weakened. In general paralysis, on the contrary, the lesions of motion generally affect the whole length of the members, and, moreover, they do not always commence in the extremities. * * * The impairment of the mental faculties in alcoholic paralysis is marked by a state of hebetude, especially after the full development of the paralytic symptoms. The memory is also greatly weakened, and the patient forgets the most important incidents in his life. He is often, however, quite conscious of his mental and physical defects. He is generally low-spirited, and often hypochondriacal. The illusions of sight are also extremely characteristic, but the patient is often aware of the unreality of these phantasms. In addition to these false perceptions of sight, which may be extremely various, the invalid is troubled with the same spectra in his sleep, and it is a peculiar feature of alcoholic intoxication, that the patient is unable to distinguish the illusions which occur during sleep from those which happen when he is awake. * * * Decided intermissions or remissions moreover occur much more frequently in alcoholic paralysis than in the general paralysis of the insane."

"*Progressive Muscular Atrophy.*—The diagnosis between muscular atrophy and paralytic insanity, when it has been ushered in without incoherence, is not an easy task. The absence of insanity at the outset of the disease, the more or less rapid development of maniacal excitement, the absence of embarrassment of speech, except perhaps towards the close of disease in one instance or at the commencement of the affection in another, are the points to which attention must be directed in our endeavour to arrive at a correct diagnosis."

Dr. Winn has, in the treatise to which we have previously referred, drawn attention to the remarkable resemblance which paralysis agitans bears to paralytic insanity. Shaking palsy often commences imperceptibly, and progresses slowly; it may commence in the head or in the arms, which may remain affected for years; after a while the paralysis extends to the legs, which become weak and tremulous, and unable to obey the will; at a more advanced period the power of speaking and eating is lost; the urine and fæces are passed involuntarily; coma at length ensues, and terminates in death. Although the two diseases are distinct, there is nevertheless a remarkable coincidence in many of the symptoms. It is also a remarkable fact, that induration of some parts of the nervous system has been discovered occasionally in both diseases.

M. Falret concludes his observations with the following inferences:

"1st. If, by the words General Paralysis, it is intended to designate a disease characterised by other phenomena than that of paralysis, then there is only one species of this affection which has, as yet, been scientifically demonstrated. 2ndly. If, on the contrary, we merely understand by this title a symptom which can supervene on various diseases, it must be admitted that there are not only two, but many kinds of General Paralysis; that there exists, for instance, the apoplectic, the epileptic, the saturnine, the alcoholic, and the atrophic forms of General Paralysis."

Nowithstanding all that has been advanced on the continent to the contrary, we are inclined to consider that the disease commonly termed in this country "the General Paralysis of the insane," is a distinct and specific form of paralysis, having a peculiar origin, progress, and termination. The question, after all, can only be determined by experience and an appeal to facts, and much more research is required before a positive opinion can be formed on the subject.

The cases which M. Falret has appended to his treatise are extremely few. They are only ten in number. Four of these were cases of General Paralysis accompanied with ideas of grandeur. Three were instances of alcoholic paralysis; one was a case of muscular atrophy; and one afforded a good example of saturnine paralysis. Perfect recovery only occurred in one instance, and that was a case of alcoholic paralysis associated with delusions and hypochondriasis. He only gives the results of three post mortem examinations, and these present nothing noticeable.

M. Falret is evidently a shrewd and clever man, and has certainly done good service to psychological science, by the industrious and methodical manner in which he has given us an exposition of the various opinions which have agitated our continental friends of late, with regard to the various and complicated phenomena of an obscure and singular malady. We could have wished that M. Falret's treatise had exhibited marks of a more extended experience; this is, however, a fault which we hope to see corrected when he next appears in print, and we trust, from some words which have fallen from him in the commencement of his thesis, that it will not be long before we have the pleasure of renewing our acquaintance with an author who, from position and capacity, is admirably qualified to investigate the nature of one of the most interesting subjects that can possibly engage the attention of the physician or the psychologist.

---

